# Delayed embolization associated with increased mortality in pelvic fracture with hemodynamic stability at hospital arrival

**DOI:** 10.1186/s13017-021-00366-z

**Published:** 2021-05-03

**Authors:** Makoto Aoki, Toshikazu Abe, Shokei Matsumoto, Shuichi Hagiwara, Daizoh Saitoh, Kiyohiro Oshima

**Affiliations:** 1 Department of Emergency and Critical Care Center, Saiseikai Yokohamashi Tobu Hospital, Yokohama, Japan; 2 Department of Emergency Medicine, Gunma University Graduate School of Medicine, Maebashi, Japan; 3 Department of Emergency and Critical Care Medicine, Tsukuba Memorial Hospital, Tsukuba, Japan; 4 Department of Health Services Research, University of Tsukuba, Tsukuba, Japan; 5 Department of Emergency Medicine, Kiryu Kosei General Hospital, Kiryu, Japan; 6 Division of Traumatology, Research Institute, National Defense Medical College, Tokorozawa, Japan

**Keywords:** Pelvic fracture, Hemodynamics, Angioembolization, Timing, Outcome

## Abstract

**Background:**

Embolization is widely used for controlling arterial hemorrhage associated with pelvic fracture. However, the effect of a delay in embolization among hemodynamically stable patients at hospital arrival with a pelvic fracture is unknown. Therefore, our aim was to investigate the association between the time to embolization and mortality in hemodynamically stable patients at hospital arrival with a pelvic fracture.

**Methods:**

A multicenter, retrospective cohort study was undertaken using data from the Japan Trauma Data Bank between 2004 and 2018. Hemodynamically, stable patients with a pelvic fracture who underwent an embolization within 3 h were divided into six groups of 30-min blocks of time until pelvic embolization (0–30, 30–60, 60–90, 90–120, 120–150, and 150–180 min). We compared the adjusted 30-day mortality rate according to time to embolization.

**Results:**

We studied 620 hemodynamically stable patients with a pelvic fracture who underwent pelvic embolization within 3 h of hemorrhage. The median age was 68 (48–79) years and 55% were male. The median injury severity score was 26 (18–38). Thirty-day mortality was 8.9% (55/620) and 24-h mortality was 4.2% (26/619). A Cochran–Armitage test showed that a 30-min delay for embolization was associated with increased 30-day (*p* = 0.0186) and 24-hour (*p* = 0.033) mortality. Mortality within 0–30 min to embolization was 0%. The adjusted 30-day mortality rate increased with delayed embolization and was up to 17.0% (10.2–23.9) for the 150–180 min group.

**Conclusion:**

Delayed embolization was associated with increased mortality in pelvic fracture with hemodynamic stability at hospital arrival. When you decide to embolize pelvic fracture patients, the earlier embolization may be desirable to promote improved survival regardless of hemodynamics.

## Background

Pelvic fracture is a type of severe trauma, with 5 to 20% of patients showing hemodynamic instability due to hemorrhage. The mortality among hemodynamically unstable patients with pelvic fracture was reported to be as high as 30 to 40% [1, 2]. Several surgical and nonsurgical interventions for pelvic fracture–related hemorrhage exist [1, 3, 4]. Of these, embolization has been widely accepted as a standard nonsurgical intervention [1]; however, this practice still has challenges. The latest version of the Resources for Optimal Care of the Injured Patient issued by the American College of Surgeons Committee on Trauma stipulates that interventional radiologists should be available within 30 min to perform an emergency embolization [5]. However, the time to embolization was reported to be prolonged even in trauma centers [6].

A reduced time to intervention is well known to be associated with decreased mortality, such as in acute coronary syndrome [7] and trauma [8]. The effect of reduced time to embolization among hemodynamically unstable patients with pelvic fracture has been described [9–12]. Earlier intervention for hemodynamically unstable patients is clinically desirable. However, the effect of earlier intervention for initially hemodynamically stable patients with pelvic fracture is unknown [11]. Because not all the hemodynamically stable patients require embolization, a delay in embolization may sometimes occur that leads to a deterioration in patients’ outcomes. Therefore, we aimed to evaluate whether a delay in embolization was associated with increased mortality in pelvic fracture with hemodynamic stability at hospital arrival.

## Methods

### Study design

This study was a multicenter, retrospective cohort study conducted using the Japan Trauma Data Bank (JTDB) data from 2004 to 2018. JTDB is a nationwide trauma registry established in 2003 by the Japanese Association for Surgery of Trauma and the Japanese Association for Acute Medicine to improve and ensure the quality of trauma care in Japan. During the study period, 291 hospitals, including 95% of all tertiary emergency medical centers in Japan, participated in the JTDB. JTDB collects 92 data elements related to patient and hospital information, such as patient demographics, physiology, abbreviated injury scale (AIS) score, injury severity score (ISS), in-hospital procedures, and survival.

### Patient selection

Patients who were directly transferred to hospital and diagnosed with pelvic fracture (existence of pelvic fracture AIS 2005 codes) were included. In addition, we targeted patients who were aged ≧16 years and who were initially treated with embolization. The following exclusion criteria for patients were defined as follows: (1) AIS grade=6 for any region, (2) underwent any surgery for hemorrhage control for associated injuries except for external fixation, (3) lacked information on vital signs of systolic blood pressure (sBP) or heart rate (HR) on hospital arrival, (4) hemodynamically unstable patients whose sBP <90 mmHg or HR >120 bpm on hospital arrival, (5) lacked information on the time from hospital arrival to embolization, (6) time from hospital arrival to embolization was over 3 h, and (7) lacked information on outcome. Regarding the time to embolization, we excluded patients who underwent pelvic embolization 3 or more hours after admission as these cases were likely non-emergency cases [1].

### Study endpoints

The primary outcome of this study was 30-day mortality, and the secondary outcome was 24-h mortality.

### Statistical analysis

Continuous variables were expressed as medians (interquartile range), while categorical variables were presented as counts and percentages. Study patients were divided into six groups according to 30-min blocks of time to pelvic embolization (0–30, 30–60, 60–90, 90–12, 120–150, and 150–180 min). Univariate analysis was performed comparing patients’ characteristics and trauma severities between the six groups. A chi-square or Fisher’s exact test was used for categorical variables, and a Mann–Whitney *U* test was used for continuous variables. A Cochran–Armitage test for trend was also performed to evaluate outcomes between the six groups. In addition, we adjusted the backgrounds of patients and the trauma severity with regard to clustering by institutions using generalized estimating equation (GEE) models with an independent working correlation matrix. Models were adjusted for age, sex, vital signs at hospital arrival, and ISS, which were selected a priori based on reported findings [9, 10]. We then used marginal standardization based on probability determined from the GEE model to estimate the adjusted 30-day mortality by the six groups. Because the 30-day mortality of the 0–30 min group was zero, we omitted these patients from the GEE model. We showed crude mortality and risk-adjusted 30-day mortality according to groups. As for sensitivity analysis, we estimated a linear relationship between the time to embolization and the 30-day mortality using a GEE model. The model was adjusted for patient demographics, such as age and sex, vital signs, and ISS, with regard to clustering by institutions. Statistical significance was defined as a two-sided *p* value < 0.05 in all statistical analyses. All analyses were performed using R software (version 3.5.2; R Foundation for Statistical Computing, Vienna, Austria) and Stata software version 15.1 (StataCorp, College Station, TX, USA).

## Results

**Fig. 1:**
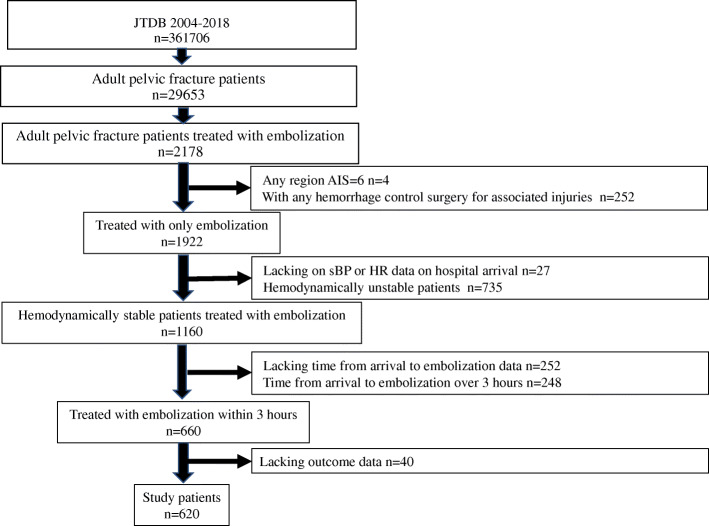
Flow chart of patients included in this study. JTDB Japan Trauma Data Bank, AIS abbreviated injury scale, sBP systolic blood pressure, HR heart rate

A total of 361,706 patients were registered in JTDB from 2004 to 2018 (Fig. 1). Of the 29,653 adult patients with a pelvic fracture, 2178 were treated with embolization. Of these, following patients were excluded: (1) AIS grade=6 for any region, *n*=4; (2) underwent any surgery for hemorrhage control for associated injuries except for external fixation, *n*=252; (3) lacking information on vital signs of sBP or HR data on hospital arrival, *n*=27; (4) hemodynamically unstable patients on hospital arrival, *n*=735; (5) lacked information on the time from hospital arrival to embolization, *n*=252; (6) time from hospital arrival to embolization was over 3 h, *n*=252; and (7) lacked information on outcome, *n*=40. Finally, 620 patients met the study criteria. Patients’ characteristics are summarized in Table 1. The median age was 68 (48–79) years and 55% were male. The median time to embolization was 101 (74–131) min and 14.5% of patients underwent embolization within 1 h. A significant difference was not observed regarding vital signs at hospital arrival and the trauma severities of pelvis AIS and ISS between the six groups. The median ISS was 26 (18–38), with ISS greater than 15 in 88.9% of patients.

**Table 1:** Patients’ characteristics of the population study

Variable	Thirty minutes to embolization							
Patients’ characteristics	Total *n*=620	0–30 *n*=12	30–60 *n*=78	60–90 *n*=152	90–120 *n*=177	120–150 *n*=118	150–180 *n*=83	*P*
Age, median	68 (48–79)	57 (42–79)	63 (47–76)	68 (45–77)	67 (48–78)	72 (54–83)	72 (51–81)	0.183
Sex, male (%)	343 (55)	5 (42)	50 (64)	78 (51)	97 (55)	69 (59)	44 (53)	0.416
Vital signs								
sBP	123 (107–141)	124 (114–127)	124 (106–141)	124 (104–146)	125 (109–143)	121 (108–140)	121 (103–138)	0.721
HR	86 (74–100)	82 (69–97)	86 (73–100)	87 (77–100)	87 (73–100)	85 (76–100)	85 (72–101)	0.933
GCS	14 (13–15)	14 (13–15)	15 (13–15)	14 (13–15)	14 (13–15)	14 (13–15)	14 (13–15)	0.966
AIS								
Head>2, *n* (%)	3 (3–4)	3 (25)	30 (39)	51 (34)	51 (29)	46 (39)	34 (41)	0.290
Chest>2, *n* (%)	3 (3–4)	1 (8.3)	28 (36)	63 (41)	71 (40)	40 (34)	25 (30)	0.132
Abdomen>2, *n* (%)	2 (2–3)	0 (0)	5 (6.4)	23 (15)	18 (10)	9 (7.6)	8 (9.6)	0.184
Pelvis AIS	4 (3–5)	4 (4–4)	4 (3–4)	4 (3–4)	4 (3–4)	4 (3-5)	4 (3-4)	0.238
ISS, median	26 (18–38)	23 (17–29)	26 (18–34)	29 (20–39)	25 (17–36)	29 (20–41)	26 (18–34)	0.213
Ps (%)	89 (73–96)	94 (78–97)	89 (79–96)	87 (65–96)	91 (78–95)	85 (60–95)	92 (76–95)	0.291

*AIS* abbreviated injury scale, *GCS* Glasgow Coma Scale, *HR* heart rate, *ISS* injury severity score, *Ps* probability of survival, *sBP* systolic blood pressure

**Fig. 2:**
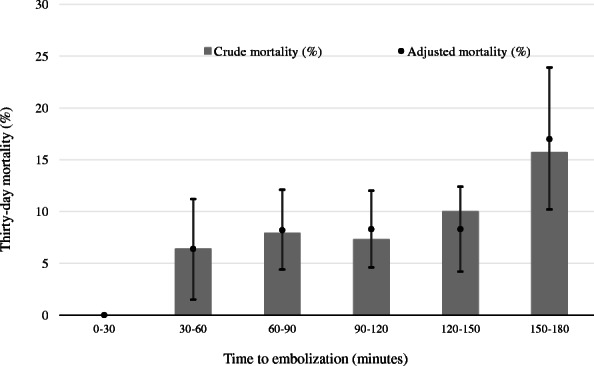
Crude 30-day and predicted 30-day mortalities, with adjustment for covariates across a range of times to embolization. Adjusted mortalities and 95% confidence intervals calculated by a generalized estimating equation model are demonstrated using bar graphs with error bars

The 30-day mortality was 8.9% (55/620) and 24-h mortality was 4.2% (26/619). Table 2 is a summary of the results of Cochran–Armitage tests for trend to evaluate outcomes. An observed trend was that a delayed time to embolization was associated with significantly increased 30-day (*p* = 0.0186) and 24-h (*p* = 0.033) mortality rates. Figure 2 shows crude mortality and the risk-adjusted 30-day mortality rate. Mortality during 0–30 min to embolization was 0%. The mortality rate increased with delayed time to embolization by up to 17.0% (10.2–23.9) for the 150–180 min group. A linear relationship was noted between time to embolization (min) and 30-day mortality in the GEE model (βcoefficient 0.0095; *p* = 0.049).

**Table 2:** Outcomes in time to embolization

Variable	Thirty minutes to angioembolization							*P*
Outcomes, n (%)	Total	0–30	30–60	60–90	90–120	120–150	150–180	
30-day mortality	55 (8.9)	0 (0)	5 (6.4%)	12 (7.9%)	13 (7.3%)	12 (10%)	13 (15.7%)	0.0186^*^
24-h mortality	26 (4.2)	0 (0)	2 (2.6)	5 (3.3)	6 (3.4)	6 (5.1)	7 (8.5)	0.033^*^

*Cochran–Armitage trend test

## Discussion

### Brief summary

This study demonstrated that delayed time to embolization was associated with increased mortality among hemodynamically stable patients with pelvic fracture. Every 30-min delay to embolization increased mortality.

### Comparison with previous studies and possible explanations for this study

This study was different in some respects from previous reports [9–11] on the assessment of the relationship between time to embolization and mortality among patients with pelvic fractures. First, we divided patients into six groups based on 30-min blocks of time although previous studies divided time to embolization by an hour [10, 11]. The availability of emergency interventional radiology was required within 30 min [5] and could be deployed within early 30-min blocks in Japan even if the patient was hemodynamically stable. Second, we focused only on patients who were hemodynamically stable at hospital arrival. Previous studies included approximately 30–40% hemodynamically unstable patients with pelvic fracture [10, 12]. Our results suggested that patients with pelvic fractures, regardless of initial hemodynamics, should be treated urgently if an intervention is considered.

One possible reason for why hemodynamically stable patients needed early intervention was that we included only embolized patients and not include patients who underwent only diagnostic angiography. Embolization is thought to be conducted in response to some sort of sign of bleeding, such as subsequent hemodynamic instability, a contrast blush on computed tomography or pelvic hematoma [12]. Another explanation may be the older age of this study’s population (median 68 years [48–79]). Kimbrell et al. reported that older patients had a high likelihood of active retroperitoneal bleeding and recommended embolization among this age group regardless of presumed hemodynamic stability [13].

### Clinical implication of this study

As we have previously stated, early identification and intervention are desirable among pelvic fracture patients with ongoing hemorrhage [14]. And if clinicians considered to conduct embolization, the earlier embolization is desirable. Delayed embolization due to a delay in decision of embolization and delay in an intervention might occur among patients who are initially hemodynamically stable. However, once patients were considered to need embolization, the patients could have continuous bleeding [3] and need prompt intervention. In such patients, as little as a 30-min delay to embolization was associated with an increased 30-day mortality. However, this study implied that we had to permit over-indicated embolization, to some extent, for hemodynamically stable patients. Early embolization may be unnecessary and a discussion about which patients really need embolization is required.

We acknowledge several limitations of this study. First, data was lacking on the indication for embolization. General indication of embolization was hemodynamic instability; however, we could not gain the information of deteriorated vital signs in further clinical course by JTDB. Second, the details of embolization for pelvic fracture were not registered in JTDB. For instance, the embolic agent and/or the embolized artery were not described. Third, the exact cause of death was unknown. We excluded the patients who underwent hemostatic procedure except for pelvis; however, we could not gain exact information of cause of death from this registry-based study. Fourth, no measure of errors in coding and data entry could be obtained. Therefore, biases introduced in this manner could not be controlled although they were likely mitigated by the multicenter design and large patient sample used.

## Conclusion

Delayed embolization was associated with increased mortality in pelvic fracture with hemodynamic stability at hospital arrival. Patients with pelvic fractures, regardless of initial hemodynamics, earliest embolization may be desirable if an intervention is considered.

## Data Availability

Not applicable

## Abbreviations

AIS: Abbreviated injury scale
GEE: Generalized estimating equation
HR: Heart rate
ISS: Injury severity score
JTDB: Japan Trauma Data Bank
sBP: Systolic blood pressure

## References

[1] Williams L, Coimbra R, Holcomb JB, Podbielski JM, Catalano R, Blackburn A, Scalea TM, Stein DM, Williams L, Conflitti J, Keeney S, Suleiman G, Zhou T, Sperry J, Skiada D, Inaba K, Williams BH, Minei JP, Privette A, Mackersie RC, Robinson BR (2016). Moore FO; AAST Pelvic Fracture Study Group. Current management of hemorrhage from severe pelvic fractures: results of an American Association for the Surgery of Trauma multi-institutional trial. J Trauma Acute Care Surg..

[2] White CE, Hsu JR, Holcomb JB (2009). Haemodynamically unstable pelvic fractures. Injury..

[3] Constantini TW, Coimbra R, Holcomb JB, Podbielski JM, Catalano RD, Blackburn A, Scalea TM, Stein DM, Williams L, Conflitti J, Keeney S, Hoey C, Zhou T, Sperry J, Skiada D, Inaba K, Williams BH, Minei JP, Privette A, Mackersie RC, Robinson BR, Moore FO (2017). AAST Pelvic Fracture Study Group. Pelvic fracture pattern predicts the need for hemorrhage control intervention-results of an AAST multi-institutional study. J Trauma Acute Care Surg..

[4] Duchesne J, Constantini TW, Khan M, Taub E, Rhee P, Morse B, Namias N, Schwarz A, Graves J, Kim DY, Howell E, Sperry J, Anto V, Winfield RD, Schreiber M, Behrens B, Martinez B, Raza S, Seamon M, Tatum D (2019). The effect of hemorrhage control adjuncts on outcome in severe pelvic fracture: a multi-institutional study. J Trauma Acute Care Surg..

[5] American College of Surgeons Committee on Trauma (2014). Resources for Optimal Care of the Injured Patient.

[6] Schwartz DA, Medina M, Cotton BA, Rahbar E, Wade CE, Cohen AM, Beeler AM, Burgess AR, Holcomb JB (2014). Are we delivering two standards of care for pelvic trauma? Availability of angioembolization after hours and on weekends increases time to therapeutic intervention. J Trauma Acute Care Surg..

[7] Cannon CP, Gibson CM, Lambrew CT, Shoultz DA, Levy D, French WJ, Gore JM, Weaver WD, Rogers WJ, Tiefenbrunn AJ (2000). Relationship of symptom-onset-to-balloon time and door-to-balloon time with mortality in patients undergoing angioplasty for acute myocardial infarction. JAMA..

[8] Remick KN, Schwab CW, Smith BP, Monshizadeh A, Kim PK, Reilly PM (2014). Defining the optimal time to the operating room may salvage early trauma deaths. J Trauma Acute Care Surg..

[9] Chou C-H, Wu Y-T, Chih-Yuan F, Liao C-H, Wang S-Y, Bajani F, Hsieh C-H (2019). Hemostasis as soon as possible? The role of the time to angioembolization in the management of pelvic fracture. World J Emerg Surg.

[10] Matsushima K, Piccinini A, Schellenberg M, Cheng V, Heindel P, Strumwasser A, Benjamin E, Inaba K, Demetriades D (2018). Effect of door-to-angioembolization time on mortality in pelvic fracture: every hour of delay counts. J Trauma Acute Care Surg..

[11] Tanizaki S, Maeda S, Matano H, Sera M, Nagai H, Ishida H. Time to pelvic embolization for hemodynamically unstable pelvic fractures may affect the survival for delays up to 60 min. Injury. 2014;45(4):738–41. 10.1016/j.injury.2013.11.007 24314873

[12] Tesoriero RB, Bruns BR, Narayan M, Dubose J, Guliani SS, Brenner ML, Boswell S, Stein DM, Scalea TM (2017). Angiographic embolization for hemorrhage following pelvic fracture: is it “time” for a paradigm shift?. J Trauma Acute Care Surg..

[13] Kimbrell B, Velmahos GC, Chan LS, Demetriades D (2004). Angiographic embolization for pelvic fractures in older patients. Arch Surg..

[14] Aoki M, Ogura T, Hagiwara S, Nakamura M, Oshima K (2019). Prediction of arterial extravasation in pelvic fracture patients with stable hemodynamics using coagulation biomarkers. World J Emerg Surg..

